# Verbesserung der Versorgung von Patientinnen und Patienten mit spastischer Bewegungsstörung nach Schlaganfall

**DOI:** 10.1007/s00115-023-01571-8

**Published:** 2023-11-21

**Authors:** John-Ih Lee, Albrecht Günther, Sebastian Paus, Georg Royl, Ute Weyen, Jörg Wissel, Kirsten E. Zeuner, Stephan Klebe

**Affiliations:** 1 Klinik für Neurologie, Medizinische Fakultät und Universitätsklinikum Düsseldorf, Heinrich-Heine-Universität Düsseldorf, Düsseldorf, Deutschland; 2 Klinik für Neurologie, Universitätsklinikum Jena, Jena, Deutschland; 3 Fachabteilung Neurologie, GFO Kliniken Troisdorf, Troisdorf, Deutschland; 4 Klinik für Neurologie, Neurovaskuläres Zentrum, Universitätsklinikum Schleswig-Holstein, Campus Lübeck, Lübeck, Deutschland; 5 BG Universitätsklinikum Bergmannsheil Bochum, Bochum, Deutschland; 6 Neurorehabilitation, Vivantes Klinikum Spandau, Berlin, Deutschland; 7 Universitätsklinikum Schleswig-Holstein, Campus Kiel, Kiel, Deutschland; 8 Klinik für Neurologie, Universitätsklinikum Essen, Essen, Deutschland

**Keywords:** Botulinumneurotoxin, Entlassungsmanagement, Rehabilitation, Schlaganfall, Spastik, Schlaganfall Nachsorge, Botulinum toxin, Discharge management, Rehabilitation, Stroke, Spasticity, Stroke aftercare

## Abstract

**Hintergrund:**

In bis zu 43 % der Fälle entwickelt sich in Folge eines Schlaganfalls eine spastische Bewegungsstörung („spastic movement disorder“, SMD). Beim Vorliegen einer funktions- oder alltagsrelevanten SMD oder zur Vermeidung von Komplikationen wird die medikamentöse Behandlung einer fokalen, multifokalen und segmentalen spastischen Tonuserhöhung mit Botulinumneurotoxin A (BoNT-A) empfohlen. Versorgungsdaten dokumentieren jedoch einen Mangel an einer leitliniengerechten Versorgung in Deutschland.

**Fragestellung:**

Ziel des berichteten Expertentreffens war es, Lösungswege aus der Fehl- und Unterversorgung für Patient*innen mit SMD zu diskutieren und eine Konsensusempfehlung zur Verbesserung der Versorgung zu formulieren.

**Methoden:**

Auf einem im April 2022 durchgeführten Konsensus-Meeting diskutierten acht Expertinnen und Experten aus den Bereichen Neurologie, Physikalische Medizin und Rehabilitation die Ursachen der Fehl- und Unterversorgung und formulierten konsentierte Lösungsansätze.

**Ergebnisse:**

Gründe der aktuellen Fehl- und Unterversorgung im SMD-Management in Deutschland umfassen ein unzureichendes Bewusstsein für SMD bei Ärztinnen und Ärzten, fehlende Behandlungskapazitäten, mangelnde Informationsübermittlung im Entlassungsmanagement und Personalmangel in spezialisierten stationären und ambulanten Therapiezentren. Das Gremium empfiehlt daher einen Patientenpfad, bei dem Betroffene mit SMD einer sachgerecht umgesetzten BoNT-A-Therapie in Kombination mit physikalischen Maßnahmen zugeführt werden.

**Diskussion:**

Der empfohlene Versorgungspfad zur Anwendung bei Patient*innen nach Schlaganfall soll Versorgungslücken schließen und damit eine leitliniengerechte Behandlung der SMD nach Schlaganfall gewährleisten.

## Abstract (en)

**Background:**

Spastic movement disorder (SMD) develops in up to 43% of cases as a sequela of stroke. In the event of a functionally relevant or daily life impairing SMD or to avoid an impending complication, the medicinal treatment of a focal, multifocal and segmental increase in muscle tone with botulinum neurotoxin A (BoNT-A) is recommended; however, treatment data reveal a lack of guideline-conform treatment with BoNT‑A in Germany.

**Objective:**

The aim of the reported expert meeting was to discuss solutions to the incorrect treatment and undertreatment of patients with SMD and to formulate consensus recommendations to improve the care situation.

**Methods:**

At a consensus meeting held in April 2022, eight experts from the fields of neurology, physical medicine and rehabilitation discussed the causes for the incorrect treatment and undertreatment and formulated consensus solution approaches.

**Results:**

Possible reasons for the current incorrect treatment and undertreatment in SMD management in Germany include insufficient awareness of SMD among physicians, a lack of treatment capacities, a lack of information transfer in discharge management as well as staff shortages in the specialized inpatient and outpatient SMD treatment centers. The committee therefore recommended a patient pathway in which affected patients with SMD are provided with correctly implemented BoNT‑A treatment in combination with physical measures.

**Conclusion:**

The recommended treatment pathway for use in stroke patients is intended to close gaps in care and thus ensure guideline-conform treatment of post-stroke SMD.

Bis zu 43 % aller Schlaganfallpatient*innen entwickeln eine spastische Bewegungsstörung („spastic movement disorder“, SMD) [22]. Die aktuelle S2k-Leitlinie der Deutschen Gesellschaft für Neurologie (DGN; gültig bis 2023) empfiehlt eine multiprofessionelle Behandlung unter Einschluss von Botulinumneurotoxin A (BoNT-A) bei Patient*innen mit SMD und einer resultierenden funktions- oder alltagsrelevanten Einschränkung oder dem Risiko von Komplikation durch die SMD [14]. Versorgungsdaten dokumentieren allerdings eine Fehl- und Unterversorgung der Betroffenen [16, 21]. Die vorliegende Arbeit soll Lösungsansätze einer Konsensusgruppe präsentieren und diskutieren, um die Fehl- und Unterversorgung zu beheben und mehr Patient*innen, die von schmerzhafter und behindernder SMD betroffen sind, den Zugang zu einer adäquaten Behandlung zu ermöglichen.

## Hintergrund und Fragestellung

Schlaganfälle führen oft zu einem Leben mit Behinderungen und Aktivitätseinschränkungen und stellen einen Einschnitt im Leben sowohl der Betroffenen als auch ihrer Angehörigen dar. Schätzungen zufolge erleiden jedes Jahr 243.000 bis 260.000 Menschen in Deutschland einen Schlaganfall [8]. Bis zu 43 % der Patient*innen entwickeln infolge des Schlaganfalls innerhalb von Wochen nach dem Akutereignis oder Monate später eine SMD, die zu einer Einschränkung der Aktivitäten und der Mobilität führen kann [22]. Die Entwicklung einer geschwindigkeitsabhängigen Tonuserhöhung als wesentliches klinisches Zeichen der SMD bereits innerhalb der ersten Woche nach einem Schlaganfall wird auf 21 % geschätzt [18], nach drei Monaten auf 19 %, nach vier Monaten auf 22 % und nach sechs Monaten auf 43 % [20]. Zwölf Monate nach einem Schlaganfall liegt, je nach Studie, bei 17–46 % der Betroffenen eine SMD vor [5, 10, 11]. Die Folgen des Schlaganfalls stellen dabei nicht nur eine Belastung für die Betroffenen, sondern auch für das Gesundheitssystem dar, insbesondere durch indirekte Kosten infolge von Erwerbsunfähigkeit, Pflegebedürftigkeit und psychosozialen Begleiterscheinungen. Allein die durch Arbeitsunfähigkeit aufgrund neuer Schlaganfälle mit SMD entstehenden jährlichen volkswirtschaftlichen Produktionsausfallkosten werden pro Person auf 23.800 € beziffert [19].

Die frühere Definition der Spastizität nach Lance als „geschwindigkeitsabhängige Muskeltonuserhöhung bei passiver Muskelstreckung“ [9] erfasst das breite klinische Erscheinungsbild der SMD nur unvollständig und wurde 2005 entsprechend umfassender als „gestörte sensomotorische Kontrolle, die aus einer Läsion des ersten Motoneurons resultiert und sich als intermittierende oder anhaltende unwillkürliche Aktivierung von Muskeln äußert“ angepasst [12]. Zur Quantifizierung einer SMD werden häufig die Ashworth-Skala [2] oder die modifizierte Ashworth-Skala (MAS; [3]) eingesetzt. Beide Skalen bilden die geschwindigkeitsabhängige Komponente der Spastizität gemäß der Definition nach Lance [9] ab. Eine Standardisierung in der Erfassung der topischen Verteilung der SMD stellt die Resistance to Passive Movement Scale (REPAS) dar [15].

Entsprechend den bis 2023 gültigen Leitlinien (LL) der DGN soll BoNT‑A zur Behandlung der fokalen, multifokalen und segmentalen spastischen Tonuserhöhung eingesetzt werden, wenn eine funktions- oder alltagsrelevante SMD oder die Möglichkeit einer Komplikation durch eine SMD besteht (z. B. die Entwicklung einer Kontraktur und mit der Spastik assoziierte Schmerzen; [14]). Trotz starkem Konsens für die Empfehlung in der LL ergab eine in 2019 publizierte Fragebogenerhebung zur Versorgungslage von Betroffenen mit SMD in Deutschland bei niedergelassenen Allgemeinmediziner*innen, dass weniger als 10 % der Patient*innen mit BoNT‑A behandelt werden [16] und nur 4 % der Patient*innen mit SMD in stationären Pflegeeinrichtungen [21]. Im Gegensatz dazu wird zu fast 100 % Physiotherapie verordnet, und etwa die Hälfte der Patient*innen analgetisch oder oral antispastisch therapiert [16]. Im Hinblick auf die durch eine SMD erheblich reduzierte Lebensqualität, gemessen anhand der EuroQoL-Skala [6], ist es dringend anzustreben, die Fehlversorgung zu korrigieren, eine leitliniengerechte Therapie mit zusätzlicher BoNT-A-Therapie zu optimieren und eine orale Medikation zu reduzieren oder abzusetzen.

## Methoden

Aufgrund der dokumentierten Fehl- und Unterversorgung von Patient*innen mit SMD sowie vor dem Hintergrund der stärkeren Einbeziehung Betroffener in Therapieentscheidungen trafen sich im April 2022 acht im Feld erfahrene Fachärzt*innen für Neurologie (davon zwei mit Zusatzqualifikation Physikalische Medizin und Rehabilitation) zu einem Konsensus-Meeting. Ziel war es, Lösungswege aus der Fehl- und Unterversorgung der SMD zu diskutieren. In zwei Breakout-Sessions wurden zunächst Gründe für die Fehl- und Unterversorgung zusammengetragen. Im Anschluss daran wurden die Ergebnisse in der Gruppe präsentiert und diskutiert. Auf der Basis der publizierten Evidenz in der S2k-LL der DGN und der konsentierten Aussagen der teilnehmenden Expert*innen wurden Verbesserungsvorschläge zum SMD-Management formuliert und abschließend konsentiert. Die Ergebnisse des Meetings wurden parallel protokolliert, anschließend in einem Manuskriptentwurf vorgelegt und mittels zweier Korrekturrunden konsolidiert und konsentiert.

## Ergebnisse

In Tab. 1 sind die von der Expert*innengruppe erarbeiteten Gründe für die aktuelle Fehl- und Unterversorgung von Schlaganfallbetroffenen mit behandlungsbedürftiger SMD zusammengefasst. Es kann geschlussfolgert werden, dass sowohl die Identifikation von Betroffenen mit Behandlungsbedarf als auch die Möglichkeiten einer angemessenen multiprofessionellen Behandlung unzureichend sind. So fehlt es in allen medizinischen Versorgungsbereichen (Krankenhäusern der Akutversorgung, Rehabilitationseinrichtungen und Arztpraxen) an einer adäquaten Vergütung und somit dem Anreiz, die Zeit aufzuwenden, um eine BoNT-A-Injektionsbehandlung flächendeckend durchzuführen. Dadurch beginnt heute in Deutschland eine BoNT-A-Therapie bei den meisten Betroffenen erst nach Abschluss einer neurologischen Rehabilitation. Des Weiteren ist bei vielen Allgemeinmediziner*innen und teilweise auch niedergelassenen Neurolog*innen die bewusste Wahrnehmung („awareness“) möglicher Probleme durch eine behandlungsbedürftige SMD gering oder gar nicht vorhanden. Dies schließt auch mögliche ernste Komplikationen einer SMD ein, z. B. assoziierte Schmerzen oder Kontrakturen.

**Table 1:** 

Setting	Hindernis
Ambulanter Bereich	Fehlendes Bewusstsein für das Problem SMD und SMD-assoziierte Komplikationen (Kontrakturen und Schmerzen)
	Geringe Akzeptanz der BoNT-A-Behandlung und Bevorzugung oraler Antispastika
	Fehlende BoNT-A-Behandler*innen, resultierend in langen Wartezeiten bis zum Termin
	Fehlende zusätzliche Vergütung, daher fehlender Anreiz, die Zeit, die für die Injektion von BoNT‑A benötigt wird, aufzuwenden
	Regressangst bei Off-label-Use
	Unzureichende Versorgung von Pflegeheimen
Stationärer Bereich	Fehlende Kapazitäten durch Personalmangel in den Spezialambulanzen der Universitätskliniken und Unterfinanzierung der Ermächtigungsambulanzen
	Screening auf SMD-Risiko auf den Schlaganfallstationen kaum implementiert
	Fehlende Informationsübermittlung von Akutkliniken in die Rehabilitationsklinik und von dort zu weiterbehandelnden niedergelassenen Neurolog*innen/Allgemeinmediziner*innen
	Fehlende interdisziplinäre Konzepte unter Einbeziehung von Neurochirurgie, Physiotherapie, Traumatologie u. a.
Insgesamt	Ungenügende lokale Vernetzung zwischen Schlaganfallstationen und BoNT-A-Ambulanz, Schlaganfallstationen und Rehaklinik, Schlaganfallstationen und Neurolog*in/Allgemeinmediziner*in, Rehaklinik und BoNT-A-Ambulanz, Neurolog*in/Allgemeinmediziner*in und BoNT-A-Ambulanz aufgrund fehlender Förderung der Kommunikation über die Sektorengrenzen hinweg
	Fehlendes Wissen über SMD-Therapieoptionen, vor allem bei Allgemeinmediziner*innen und z. T. bei niedergelassenen Neurolog*innen
	Fehlende Einbindung von Physio‑, Ergotherapeut*innen, Orthopädietechniker*innen und Redressionsspezialist*innen (z. B. Hilfsmittelanbieter*innen)
	Fehlendes bzw. mangelhaftes Entlassungsmanagement bezüglich SMD-Risiko und Weiterversorgung der Patient*innen
	Unzureichende Aufklärung von Patient*in und Angehörigen zu SMD
	Mangelnde Unterstützung intersektoraler Versorgungsforschung auf dem Gebiet SMD

*BoNT* Botulinumneurotoxin, *SMD* spastische Bewegungsstörung

Die genannten Aspekte treffen auf eine meist unzureichende lokale Vernetzung der Behandelnden, die nach LL als erforderlich eingestuft wird (Comprehensive Care, analog zur Versorgung auf einer Schlaganfallstation oder in der Neurorehabilitation; [14]). Das Netzwerk zur Betreuung von SMD-Betroffenen sollte im Idealfall Krankenhäuser der Erstversorgung nach einem Schlaganfall, Rehabilitationsmediziner*innen bzw. Neurorehabilitationseinrichtungen, Allgemeinmediziner*innen, niedergelassene spezialisierte Neurolog*innen und BoNT-A-Ambulanzen einbeziehen. Dabei entsteht ein Informationsdefizit oft schon im Entlassungsmanagement aus dem klinischen Bereich (Schlaganfallstation, Neurorehabilitation), sodass es nicht verwunderlich ist, dass viele Patient*innen auch bei drohenden Komplikationen oder behindernden Symptomen einer SMD nicht leitliniengerecht behandelt werden oder der Ausbildung einer SMD-Entwicklung nicht frühzeitig entgegengewirkt wird.

## Diskussion von Lösungswegen und Konsensus

**Figure 1:**
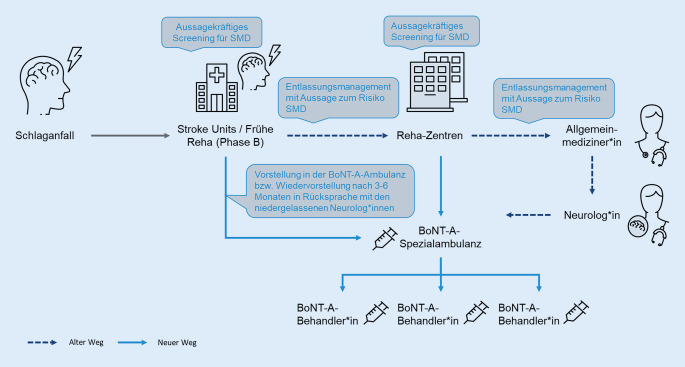

Da der bisher gelebte Patientenpfad zur Behandlung einer SMD häufig zu einer Fehl- und Unterversorgung führt, schlagen die konsentierenden Mitwirkenden einen neuen Behandlungspfad vor, um die Behandlung zu verbessern (Abb. 1). Ziel ist, dass SMD-Betroffene bei entsprechender Indikation eine höhere Wahrscheinlichkeit haben, eine BoNT-A-Therapie zu erhalten.

Eine SMD sollte nach Empfehlung der LL der DGN so früh wie möglich behandelt werden, um Komplikationen zu verhindern. Daher sollten alle Patient*innen nach einem Schlaganfall, die ein Risiko für eine behandlungsbedürftige SMD tragen, durch ein aussagekräftiges Screening identifiziert und einer LL-gerechten Therapie zugeführt werden. Falls es sich dabei um eine lokalisierte (fokale, multifokale oder segmentale) SMD handelt, sollte eine BoNT-A-Behandlung mit einer begleitenden Physio- und Ergotherapie eingeleitet werden.

Das von der Konsensusgruppe vorgeschlagene Screening sollte bereits in der Akutversorgung (Schlaganfallstation) innerhalb der ersten sieben Tage nach dem Schlaganfall oder in der neurologischen Rehabilitation anhand publizierter Prädiktoren der SMD-Entwicklung [4, 23] erfolgen. Für die Beurteilung einer geschwindigkeitsabhängigen Erhöhung des Muskeltonus steht mit der REPAS ein standardisiertes und validiertes klinisches Instrument mit etablierten Cut-off-Werten zur Verfügung [15]. Eine Kombination von klinischen Parametern und bildgebenden Verfahren bietet die Möglichkeit für eine frühe Identifizierung [17], ist jedoch im klinischen Alltag aufgrund mangelnder Ressourcen häufig nicht anwendbar. Als klinische Cut-off-Parameter wurden in einer Studie eine National Institutes of Health Stroke Scale (NIHSS) > 2, modifizierte Rankin-Skala (mRS) > 2 und Mini-Mental-Status-Test < 27 diskutiert, wobei eine relevante Parese zwar Voraussetzung, aber kein Prädiktor für das Auftreten einer SMD war [4]. In anderen Studien wurden sowohl funktionell relevante Paresen mit Auswirkungen auf Alltagsaktivitäten (ADL) als auch eine frühe Tonuserhöhung in mehr als einem Gelenk als gute Prädiktoren identifiziert [23].

Die bei stationärer Aufnahme und Entlassung erhobenen Parameter mRS und NIHSS sollten im Arztbrief dokumentiert werden, sowohl zum Zeitpunkt der Verlegung aus der Akutklinik in eine Rehabilitationseinrichtung als auch bei der Weiterleitung zum/zur Allgemeinmediziner*in oder zum/zur Fachärzt*in für Neurologie oder Physikalische Medizin und Rehabilitation. Darüber hinaus sollte das individuelle Risiko für die Ausbildung einer SMD in der Folge eines Schlaganfalls in Form einer Ampel dargestellt werden, wobei die folgenden Cut-off-Werte eingesetzt werden können:

Farbe „Rot“: Ein hohes Risiko. Es liegt ein akuter physikalischer und medikamentöser Behandlungsbedarf vor. Rot wird bei einem mRS-Wert von 4 oder 5 und einem NIHSS-Wert ≥ 8 mit spastischer Tonuserhöhung mit MAS ≥ 2 in mehr als einem Gelenk kodiert und beschreibt behindernde und klinisch relevante SMD-Symptome, gegebenenfalls durch REPAS bestätigt. Farbe „Gelb“ zeigt ein mittleres Risiko an: Aktuell besteht ein physikalischer Behandlungsbedarf, und im Verlauf – nach spätestens sechs Monaten – muss eine Kontrolle erfolgen. Gelb ist definiert als mRS > 2 und NIHSS > 2 sowie Fehlen einer signifikanten spastischen Tonuserhöhung (MAS < 2). Ein geringes Risiko einer SMD wird durch die Farbe „Grün“ kodiert, bei einem mRS ≤ 2 und NIHSS ≤ 2 ohne spastische Tonuserhöhung.

Es ist zu betonen, dass es sich bei dieser Ampel nicht um eine durch eine prospektive Studie abgesicherte Empfehlung und somit nicht um einen validierten Score handelt. Vielmehr ist sie als Konsensusstatement zu verstehen, die das Risiko erkennbar macht, eine SMD zu entwickeln. Ziele der Maßnahme sind, Risikopatient*innen zu erfassen, damit sie in regelmäßigen klinischen Kontrollen (mindestens alle 6 Monate) durch im Feld erfahrene Neurolog*innen und/oder Ärzt*innen für Physikalische Medizin und Rehabilitation untersucht werden, eingebettet in ein multiprofessionelles Netzwerk. Bei entsprechender Indikationsstellung sollen sie schnell einer spezifischen Behandlung zugeführt werden [14]. Zur Überprüfung dieser Vorgehensweise sollten prospektive Untersuchungen folgen, um die Prädiktoren weiter zu schärfen.

Patient*innen, die bereits auf der Schlaganfallstation bzw. innerhalb der ersten sieben Tage nach dem Schlaganfall eine SMD entwickeln, sollen sofort angemessen physikalisch und medikamentös entsprechend der LL der DGN behandelt werden. Hingegen sollten Patient*innen, die nicht von der Akutklinik in die Rehabilitationsklinik weitergeleitet werden, im Entlassungsbrief an den weiterbehandelnden Arzt bzw. an die weiterbehandelnde Ärztin den Hinweis erhalten, dass innerhalb der nächsten sechs Monate eine neurologische Nachsorge bei einem spezialisierten Behandelnden des spastischen Syndroms erfolgen sollte. Entwickelt sich während der neurologischen stationären oder ambulanten Rehabilitation eine SMD, sollte dies anhand der REPAS dokumentiert, bei Relevanz (MAS ≥ 2) behandelt und im Entlassungsbrief vermerkt werden. Zudem sollen Patient*innen sowie ihre Angehörigen Zugriff auf entsprechende Aufklärungsmaterialien und eine Liste von ambulanten, in erreichbarer Nähe zum Wohnort verfügbaren multiprofessionellen Behandelnden erhalten.

Die Therapie mit BoNT‑A sollte bei Bedarf auch wiederholt zur Verfügung stehen. Eine dauerhafte physikalische Therapie ist bei Patient*innen mit chronischer SMD in Deutschland bei den Kostenträgern unumstritten. Hingegen existiert für den ambulanten Sektor und die Rehabilitationsmedizin aktuell keine leistungsspezifische EBM-Ziffer, über die Fachärzt*innen die BoNT-A-Therapie bei Patient*innen mit SMD der oberen oder unteren Extremität nach einem Schlaganfall abrechnen könnten [7]. Ausnahmen stellen die Bundesländer Bayern (Sonderziffer für BoNT-A) und Baden-Württemberg (Facharztvertrag mit dem Medi Verbund, über den BoNT‑A abgerechnet werden kann) dar (Stand 7/2023). Eine diesbezügliche leistungsspezifische EBM-Ziffer für alle Bundesländer wird daher von der Konsensusgruppe empfohlen. Aufgrund der Häufigkeit einer behandlungsbedürftigen SMD nach Schlaganfall ist es zudem zwingend notwendig, die Kapazitäten in den Ambulanzen zu erhöhen und neue BoNT-A-Behandler*innen auszubilden.

Bei einer Indikation zur BoNT-A-Behandlung ist bei kleinen und tiefer gelegenen Muskeln zur Verbesserung der Zielgenauigkeit der Injektionen eine Kontrolltechnik zu bevorzugen (Ultraschall‑, Elektromyographie- oder Elektrostimulationstechnik; [14]). Um die BoNT-A-Ambulanzen der Universitäts‑, Akut- und Rehabilitationskliniken zu entlasten und so gegebenenfalls Wartezeiten dort zu verkürzen, ist eine unmittelbare Weiterleitung der behandlungsbedürftigen SMD-Patient*innen zu qualifizierten niedergelassenen BoNT-A-Behandler*innen sinnvoll. Dieser Idee folgend wurde das sog. SMART-Behandler*innen-Konzept [24] entwickelt. Es soll die Kapazitäten für BoNT-A-Injektionen erhöhen, indem niedergelassene Neurolog*innen lernen, BoNT-A-Injektionen ohne bildgebende Verfahren durchzuführen, basierend auf anatomischen Landmarken. Statt einzelne Muskeln zu injizieren, werden mehrere Muskeln ähnlicher Funktion zu einer Muskelgruppe zusammengefasst und mit BoNT‑A infiltriert. Wirksamkeit und Sicherheit des SMART-Konzeptes werden aktuell in der SMART-Studie (NCT05224349) untersucht [24]. Da ihre Ergebnisse noch nicht vorliegen und die Bewertung nur im Vergleich zu Ergebnissen gezielter Behandlungen zu bewerten ist, konnte zum SMART-Behandler*innen-Konzept noch kein Konsens in der Gruppe erreicht werden.

Der Erfolg der Schlaganfallnachsorge im Bereich der SMD-Versorgung ist von der Vernetzung und dem kommunikativen Austausch zwischen beteiligten professionellen Akteuren untereinander, Betroffenen und Angehörigen abhängig. Dieser Austausch ist durch den aktuellen Personalmangel im deutschen Gesundheitswesen zusätzlich eingeschränkt. Um diese Unterversorgung, auch bezüglich des Screenings von Risikopatient*innen zumindest zeitweise zu überbrücken, könnte der Austausch von Informationen über Risikopatient*innen digital erfolgen. Hierfür wurde durch eine Zusammenarbeit der Bayerischen TelemedAllianz und den Schön Kliniken Bad Aibling die sog. „Spastik App“ entwickelt, die Patient*innen unterstützt, Frühzeichen einer sich entwickelnden SMD zu erkennen und dementsprechend den Allgemeinmediziner bzw. die Allgemeinmedizinerin oder den behandelnden Neurologen bzw. die Neurologin zu kontaktieren [1]. Darüber hinaus hat sich eine digitale Version der Post-Stroke Checklist (PSC) mit dem Parameter Steifheitsentwicklung als aussagekräftiges Tool für das Monitoring erwiesen [13].

Die Konsensusempfehlungen der Expert*innengruppe, die sich aus der Diskussion der Ergebnisse im Rahmen des Advisory Boards ergeben haben, sind in Tab. 2 zusammengefasst.

**Table 2:** 

Setting	Empfehlung
Allgemein	Neurologische Betreuung für alle Schlaganfallpatient*innen
	Alle Folgesymptome in der Schlaganfallnachsorge beachten
	Frühestmögliche Identifizierung von Patient*innen durch ein aussagekräftiges Screening für die Entstehung einer SMD
	Frühe Behandlung einer spastischen Bewegungsstörung
Risiko-Assessment	Risiko-Assessment mittels einer Risiko-Ampel für die Entwicklung und die Erfassung einer SMD mit folgenden Cut-offs: – Rot: mRS 4 oder 5 und NIHSS ≥ 8 und MAS ≥ 2 in ≥ 2 Gelenken – Gelb: mRS > 2 und NIHSS > 2 und MAS < 2 – Grün: mRS ≤ 2 und NIHSS ≤ 2 und MAS 0 in allen Gelenken
Diagnose einer SMD	Erhebung der REPAS zu Beginn und zum Ende der neurologischen Rehabilitation
	Prüfung des Niveaus der geschwindigkeitsabhängigen Tonuserhöhung (AS oder MAS) durch den überweisenden Arzt/die Ärztin, um eine gezielte Überweisung an die BoNT-A-behandelnden Praxen oder Spezialambulanzen zu ermöglichen
	Vermeidung von Sekundärkomplikationen durch frühe BoNT-A-Therapie bei signifikanter spastischer Tonuserhöhung (AS oder MAS ≥ 2)
Schlaganfallstation	Unmittelbare physikalische und medikamentöse Behandlung von Patient*innen, die bereits auf der Stroke-Unit bzw. innerhalb der ersten 7 Tage eine nach DGN-LL S2k behandlungsbedürftige SMD entwickelt haben
	Patient*innen ohne neurologische Rehabilitation: Hinweise im Entlassungsbrief an den weiterbehandelnden Arzt/die Ärztin, dass sich der/die betroffene Patient*in/SMD-Risiko-Patient*in innerhalb der nächsten 6 Monate zur neurologischen Nachsorge bei einem/einer spezialisierten Behandler*in oder spezialisierten Zentrum für die Behandlung der SMD vorstellen sollte (Empfehlung der DGN-LL)
Rehaklinik	Hinweise auf die Entwicklung einer SMD sollten beachtet werden und zu einer spezifischen physikalischen und DGN-LL-gerechten medikamentösen Therapie führen und im Entlassungsbrief enthalten sein
	Den Angehörigen und Patient*innen soll eine Liste mit spezialisierten Zentren für die Behandlung der SMD ausgehändigt werden, inkl. BoNT-A-Behandler*innenliste
	Im Fall einer positiven Reevaluation auf lokalisierte SMD sollte eine direkte Empfehlung im Rahmen des Entlassungsmanagements zu einer umgehenden Vorstellung bei einem BoNT-A-Behandelnden führen
BoNT-A-Therapie	Die multiprofessionelle physikalische und medikamentöse Therapie der SMD, z. B. mit BoNT‑A, kann eine repetitive Behandlung sein
	Erhöhung der Kapazitäten in den BoNT-A-Ambulanzen bzw. Ausbildung neuer BoNT-A-Behandler*innen
	Schaffung einer leistungsspezifischen EBM-Ziffer in allen Bundesländern, über die befähigte Ärzte*innen die BoNT-A-Therapie pro Behandlung bei Patient*innen mit SMD der oberen oder unteren Extremität nach einem Schlaganfall abrechnen können
Digitale Hilfsmittel	Digitale Hilfsmittel zur Überbrückung der derzeitigen personellen Unterversorgung stellen innovative Ansätze zur Kommunikation und Identifikation von SMD-Patient*innen dar
	Ein Monitoring der SMD im Langzeitverlauf ist mittels digitaler Version z. B. der Post-Stroke Checklist möglich
Ausblick	Die Behandlung der SMD sollte eine sog. Comprehensive Care darstellen und im Netzwerk eines multiprofessionellen Behandlungskonzeptes umgesetzt werden
	Schaffung einer bundesweiten EBM-Ziffer für die Koordination eines multiprofessionellen Teams (Physio- und Ergotherapie) der Spastizitätsbehandlung (z. B. abrechenbar 1‑mal im Quartal für Hausärzte*innen, Neurologen*innen)
	Die Kooperation mit den bestehenden neurovaskulären Netzwerken oder ein Versorgungskonzept für Patient*innen mit SMD nach Schlaganfall stellen mögliche Entwicklungsstränge einer flächendeckenden Versorgung der behindernden SMD dar und sollten in Innovationsfondprojekten evaluiert werden
	Notwendigkeit der Unterstützung intersektoraler Versorgungsforschung auf dem Gebiet SMD

*BoNT* Botulinumneurotoxin, *mRS* modifizierte Rankin-Skala, *NIHSS* National Institutes of Health Stroke Scale, *REPAS* REsistance to PAssive movement Scale, *SMD* spastische Bewegungsstörung

## Fazit für die Praxis

- Patient*innen mit einer fokalen, multifokalen oder segmentalen funktions- oder alltagsrelevanten SMD oder dem Risiko für eine Komplikation durch die SMD sollten leitliniengerecht (LL der DGN) mit BoNT‑A behandelt werden.
- Durch einen Patient*innenpfad sollen Versorgungslücken geschlossen und eine höhere Rate einer leitliniengerechten Behandlung von SMD-Patient*innen gefördert werden.
- Voraussetzung dafür ist nach Einschätzung der Konsensusgruppe ein möglichst frühes Screening auf ein SMD-Risiko (möglichst innerhalb von sieben Tagen), um eine frühe LL-gerechte Behandlung zu gewährleisten.
- Zur besseren Einschätzung des SMD-Risikos formulierte die Konsensusgruppe sog. „Cut-off-Werte“ für eine leichtere Interpretation des frühen Screenings der SMD-Risiken (Parameter mRS, NIHSS und MAS).
- Entsprechende Informationen zum SMD-Risiko sollten konsequent im Entlassungsbrief vermerkt und so an die Weiterbehandelnden geleitet werden.
- Die Konsensusgruppe empfiehlt eine leistungsspezifische EBM-Ziffer in allen Bundesländern, über die befähigte Ärzte*innen die BoNT-A-Therapie pro Behandlung bei Patient*innen mit SMD der oberen oder unteren Extremität nach einem Schlaganfall abrechnen können und eine bundesweite EBM-Ziffer für die Koordination eines multiprofessionelles Teams (Physio- und Ergotherapie) der Spastizitätsbehandlung (z. B. abrechenbar 1‑mal im Quartal für Hausärzte*innen, Neurologen*innen).
- Zur Überbrückung der derzeitigen personellen Unterversorgung können innovative Strategien, z. B. digitale Hilfsmittel, genutzt werden.
